# Sleep Disorders Are Associated with Mental Health, Quality of Life and Stigma in an Italian Cohort of People Living with HIV

**DOI:** 10.3390/brainsci15040332

**Published:** 2025-03-23

**Authors:** Valentina Massaroni, Valentina Delle Donne, Francesca Lombardi, Arturo Ciccullo, Valentina Iannone, Pierluigi Francesco Salvo, Daniela Pia Rosaria Chieffo, Valentina Arcangeli, Nicoletta Ciccarelli, Simona Di Giambenedetto

**Affiliations:** 1 Department of Health Science and Public Health, Faculty of Medicine and Surgery, Catholic University of the Sacred Heart, 00168 Rome, Italy; valentina.massaroni@unicatt.it; 2 Clinical Psychology Unit, Fondazione Policlinico Universitario A. Gemelli, IRCCS, 00168 Rome, Italy; valentina.delledonne@guest.policlinicogemelli.it (V.D.D.); danielapiarosaria.chieffo@policlinicogemelli.it (D.P.R.C.); valentina.arcangeli@policlinicogemelli.it (V.A.); 3 UOC Infectious Diseases, Fondazione Policlinico Universitario A. Gemelli, IRCCS, 00168 Rome, Italy; francesca.lombardi@policlinicogemelli.it (F.L.); valentina.iannone90@gmail.com (V.I.); pierluigifsalvo@gmail.com (P.F.S.); simona.digiambenedetto@unicatt.it (S.D.G.); 4 UOC Infectious Diseases, Ospedale San Salvatore, 67100 Aquila, Italy; arturo.ciccullo@gmail.com; 5 Department of Safety and Bioethics, Infectious Diseases Institute, Catholic University of the Sacred Heart, 00168 Rome, Italy; 6 Department of Woman, Children and Public Health, Catholic University of Sacred Heart, 00168 Rome, Italy; 7 Department of Theoretical and Applied Sciences, eCampus University, 22060 Novedrate, Italy

**Keywords:** HIV, sleep disorders, mental health, quality of life, stigma

## Abstract

**Objectives**: The aim of this study was to assess sleep quality in people living with HIV (PLWH), and to examine how the sleep sphere interacts with mental health, quality of life and internalized stigma. **Methods:** A total of 250 PLWH were consecutively enrolled during routine outpatient visits. Each participant completed a 67-item questionnaire. Sleep disturbances were measured using the Pittsburgh Sleep Quality Index (PSQI). The Depression, Anxiety and Stress Scale (DASS-21) was used to measure the mental health status. The Short Form 12 (SF-12) questionnaire was used to measure participants’ quality of life. Internalized HIV-related stigma was evaluated using the modified six-item internalized AIDS-related stigma scale. **Results**: Many of the PLWH were male (69.2%) and the time between HIV diagnosis and first antiretroviral therapy (ART) was over 10 years (69.2% and 64%, respectively). The PSQI component most-cited as problematic by PLWH was habitual sleep efficiency (52.4%). In multivariate analysis models, a higher mean in the PSQI total score was significantly associated with internalized stigma (mean change 1.10), depression (mean change 6. 20), anxiety (mean change 12.15), stress (mean change 6.24), physical (mean change 7.54) and mental (mean change 3.56) quality of life, health status (mean change −6.04), ART adherence (mean change −5.08) and physical activity (mean change −6.20). **Conclusions**: Our results confirm the role of sleep quality in both mental and physical health and suggest that sleep disorders might also be a significant indicator of psychosocial challenges faced by PLWH.

## 1. Introduction

Antiretroviral therapy (ART) has improved the quality of life of people living with HIV (PLWH). It has also increased the average life expectancy of this population. In Italy, an estimated 52.7 percent of PLWH are over the age of 50 [1]. However, many psychosocial challenges, such as internalized stigma [2], and mental health problems (e.g., depression, anxiety, substance abuse, and risk of suicide) [3] can impair the quality of life of PLWH. In this population, anxiety, depression and perceived internalized stigma are common and can interfere with physical, mental, and emotional well-being, ultimately affecting sleep quality [4]. Furthermore, sleep disturbances can also impair quality of life and cognitive functions, with a negative impact on treatment adherence [5,6].

From 40 to 80% of PLWH experience sleep disorders [7,8]. This percentage varies depending on patients’ sociodemographic characteristics and the type of study protocol adopted [9]. The most common sleep disorder in PLWH appears to be insomnia [6]. Other sleep disorders include longer-than-average sleep duration [7], restless leg syndrome [9], and obstructive sleep apnea [10], all of which can affect the quality of life of PLWH [11].

Poor sleep quality can have a negative impact on psychological health and lead to an altered immune system and impaired cell growth, causing deterioration of neuronal connections [12]. It is also associated with poor performance, work injuries, traffic accidents, increased use of health care services, and suicidal ideation [13,14]. Conversely, good sleep quality is associated with lower severity of HIV-related symptoms, optimal cognitive functioning—particularly in terms of memory—and better adherence to ART [12]. Therefore, by identifying and assessing sleep-related problems, it is possible to improve the immune system and slow down the progression of the disease [14].

Sleep disorders are multifactorial in nature. Among the factors that can lead to poor sleep quality in PLWH, there are long disease duration [15], a treatment regimen containing efavirenz [5], an unsatisfactory lifestyle [16], poor social support, internalized stigma [17] and inappropriate sleep-related behaviors [18,19].

With regard to internalized stigma, HIV-related stigma includes negative attitudes, behaviors and judgments directed toward PLWH [20]. According to UNAIDS reports [21], at the societal level, stigma acts as a barrier, hindering access to care services in terms of prevention and treatment and resulting in non-adherence to therapy. At the individual level, Earnshaw and Chaudoir [22] identified three mechanisms through which stigma operates in the experiences of PLWH. The first is enacted stigma, which refers to experiences of others’ prejudice and discrimination. The second is anticipated stigma, which refers to the expectation of encountering prejudice and discrimination in the community due to one’s health status. The third is internalized stigma, which refers to negative beliefs and self-perceptions that PLWH develop about themselves due to their HIV status.

PLWH who experience high levels of internalized stigma report fear and anxiety about others discovering their illness, social isolation and decreased psychological well-being [23]. This condition leads to a decrease in sleep quality [24]. Thus, it is likely that internalized stigma is associated with poor sleep quality in PLWH as a result of increased social isolation and symptoms of depression [22,25].

In Italy, studies on sleep quality in PLWH have primarily focused on the relationship between sleep disturbances and comorbidities (e.g., cardiovascular risk factors and mood disorders) [26,27] or have considered sleep disturbances as undesirable effects of medications [28,29]. Fewer studies have investigated the impact of psychological and social variables, such as internalized stigma, on sleep quality in PLWH.

Considering these findings, the purpose of this study was to assess sleep quality in an Italian cohort of PLWH and to examine the interaction of sleep quality with mental health, quality of life, and internalized stigma.

## 2. Materials and Methods

### 2.1. Participants

Two hundred and fifty PLWH were consecutively enrolled from April to June 2023 at the Infectious Disease Institute, Fondazione Policlinico Universitario A. Gemelli IRCCS, Rome, which is one of the main referral centers for all PLWH from southern central Italy. During routine outpatient visits, patients were asked to complete an anonymous paper questionnaire after the referring infectious disease physician explained the purpose and aims of the research. All participants were volunteers and received no financial remuneration for their participation. Exclusion criteria were age <18 years and difficulty with the Italian language.

### 2.2. Procedure

Each participant completed a 67-item questionnaire. The following sociodemographic data were collected: sex (male, female), age range (18–40, 40–60, >60), education (lower secondary school, upper secondary school, university degree), employment status (unemployed, employed or retired). The following clinical data were also collected: time from HIV diagnosis (<5 years, 5–10 years, >10 years), time of first ART (<5 years, 5–10 years, >10 years), HIV-RNA < 50 copies/mL (yes or no) and adherence to ART in the previous month (i.e., poor, good or excellent). A self-report judgment concerning their own state of health was also collected, i.e., poor, good or excellent. The respondents were asked about their engagement in physical activity (i.e., never, occasionally, regularly).

All procedures were conducted in accordance with the ethical standards of the institutional and national research committee and with the 1964 Helsinki Declaration and its later amendments or comparable ethical standards. The study was approved by the Ethics Committee of the Catholic University of the Sacred Heart, Rome, Italy (Protocol ID 5250).

### 2.3. Sleep Quality

The Italian version of the Pittsburgh Sleep Quality Index (PSQI) was used to assess sleep quality [29]. The questionnaire consists of 19 self-assessment questions that investigate various aspects of sleep quality, including an estimate of sleep duration and the latency and frequency of various sleep-related problems. The 19 questions are grouped into 7 areas, each of which has equal weight and can produce a score from 0 to 3. The areas are as follows: subjective sleep quality, sleep latency, sleep duration, habitual sleep efficiency, disturbed sleep, sleep medication use, daily dysfunction. The scores obtained in each area are summed in order to obtain the overall PSQI score, which ranges from 0 to 21. A higher score indicates poor sleep quality [30].

### 2.4. Mental Health Measure

The Depression, Anxiety and Stress Scale (DASS-21) was used to measure mental state [31]. The DASS-21 is a set of three self-report scales used to measure emotional states of depression, anxiety and stress. The first subscale (DASS-Depression) measures depressed mood and loss of self-esteem. The second scale (DASS-Anxiety) measures fear and anticipation of negative events. The third subscale (DASS-Stress) measures a persistent state of hyperarousal and low frustration tolerance. A high score indicates the presence of an emotional state of distress [32]. In each subscale, symptoms are categorized as normal, mild, moderate, severe and extremely severe [33]. In our study analyses, we also used the scores of each subscale divided into two categories: normal and pathological. The pathological score encompasses mild, moderate, severe and extremely severe symptoms.

### 2.5. Quality of Life Measure

The Short Form 12 (SF-12) questionnaire was used to measure quality of life. This questionnaire consists of 12 items that are part of the 8 subscales of the 36-item Short Form Health Survey (SF-36) [34]. Of the 12 items that make up the SF-12, 6 measure physical health status and 6 measure mental health status. For both physical and mental health status, the score varies on a scale ranging from 0 (poor) to 100 (excellent). Higher scores are associated with higher quality of life [35].

### 2.6. Stigma Measure

Internalized HIV-related stigma was measured using the modified six-item internalized AIDS-related stigma scale [36]. This scale measures several aspects of self-perception related to living with HIV. For example, it investigates the emotions PLWHs may feel, probing how they may feel dirty, guilty, worthless and ashamed because of their HIV status. It also investigates more behavioral aspects related to living with the disease, such as whether patients feel they have difficulty talking about their health status or hide their HIV from others. The scale consists of six statements (1—It is difficult to tell people about my HIV infection; 2—I hide my HIV status from others; 3—I feel guilty that I am HIV-positive; 4—I am ashamed that I am HIV-positive; 5—Being HIV-positive makes me feel dirty; 6—I sometimes feel worthless because I am HIV-positive). For each statement, the patient can say they agree with what is expressed (agreement should be given 1 point), disagree (disagreement should be given 0 points) or neither agree nor disagree (neutrality is worth 0.5 points). The scores obtained on the six statements are added together. Scores range from 0 (low stigma) to 6 (high stigma) [37]. In our analyses, we categorized the internalized stigma scale into low internalized stigma (scores from 0 to 2) and high internalized stigma (scores from 3 to 6).

### 2.7. Statistical Analysis

Descriptive statistical analysis was used to summarize the sociodemographic characteristics and HIV-related clinical variables of the study population. Specifically, qualitative variables were expressed as absolute frequencies and percentages. Quantitative variables were described as median and interquartile ranges (IQR) or means and standard deviations (SD). The Kolmogorov–Smirnov test was used to determine whether our data differed from normal distribution. Given the non-normal distribution, we used the nonparametric Mann–Whitney U test to compare rank means between groups, and the Kruskal–Wallis test for comparisons among multiple groups. We also ran multivariate linear regression analyses to explore the associations between the PSQI total score and the demographic and clinical characteristics, the DASS-21, the SF-12 and internalized stigma scores, adjusting for those variables that showed a *p* value < 0.005 in the univariate analysis. The univariate analysis was conducted primarily to screen potential predictors for inclusion in the multivariate models. In univariate analyses, we report median differences for non-parametric tests (Mann–Whitney U). In contrast, for multivariate analyses using regression models, we report mean change, indicating the estimated variation in the dependent variable per unit increase in the predictor variable.

Variables were selected based on a combination of statistical significance (*p* < 0.005 in univariate analysis) and theoretical relevance in the context of previous literature and clinical importance. In particular, even if some variables did not reach statistical significance in univariate analysis, they were retained in the multivariate model if deemed conceptually relevant (e.g., education as a key socioeconomic factor). This approach ensures that our model captures both statistically significant and clinically meaningful associations.

To evaluate the overall fit of the multivariate regression models, we calculated model fit indices, including the coefficient of determination (R^2^), the adjusted R^2^, the Akaike Information Criterion (AIC) and the Bayesian Information Criterion (BIC). These indices provide insights into the explanatory power of the model and its parsimony, ensuring that the selected predictors contribute meaningfully to the explained variance. High collinearity among predictors was assessed using the Variance Inflation Factor (VIF) and adjustments were made where necessary to optimize model interpretability. High VIF values (above 10) indicate severe collinearity, which may distort regression estimates. Given the strong correlation among DASS-21 Depression, Anxiety and Stress, we applied Principal Component Analysis (PCA) to reduce them into a single Psychological Distress Factor. This transformation was performed using standardized scores to maintain comparability. The revised model was then reassessed for collinearity using VIF.

Due to collinearity among the psychosocial variables, several multivariate models were run. In the multivariate analysis, mean change estimates are reported in relation to a reference group. The reference category was defined based on theoretical and clinical relevance. Specifically, for categorical variables, the reference group was set as the category theoretically expected to have a lower burden (e.g., male participants, individuals with low stigma, those classified as non-pathological in DASS-21). For continuous variables, the mean change reflects the expected variation per unit increase in the predictor variable. A two-tailed *p*-value <0.05 was considered statistically significant. All analyses were carried out using the SPSS version 21.0 software package (SPSS Inc., Chicago, IL, USA).

We ensured that the interpretation of all instrument scores was aligned correctly in the statistical analysis. Specifically, higher scores on the PSQI indicate poorer sleep quality, whereas higher scores on the SF-12 reflect better quality of life. To facilitate interpretation, the direction of effect estimates was carefully considered; positive mean changes in PSQI scores represent worsening sleep quality, whereas positive mean changes in SF-12 scores indicate better quality of life. This approach ensures consistency in the interpretation of associations.

## 3. Results

### 3.1. Demographic and Clinical Characteristics

We enrolled 250 PLWH on ART. The majority were males (69.2%, n = 173) who ranged in age from 41 to 60 years (49.6%, n = 124) and who had an upper secondary school degree (51.2%, n = 128) and were employed (61.6%, n = 154). Most patients had received an HIV diagnosis and had started ART more than ten years before (69.2%, n = 173 and 64.0%, n = 170, respectively). Most patients had a viremia inferior to 50 copies (92.8%, n = 232). Good health status was reported by 125 patients (i.e., 50%) and 120 (48.0%) had excellent adherence. Occasional physical activity was reported by 115 patients (46.0%). Demographic and clinical characteristics of the sample are shown in Table 1.

### 3.2. Sleep Quality Evaluation

According to the PSQI, the fourth component, i.e., “habitual sleep efficiency” was the most difficult one for the patients. In fact, more than half of them (52.4%, n = 131) reported having great difficulty with this component. The complete results obtained for each component are shown in Table 2.

### 3.3. Multivariate Linear Regression Analysis

In the univariate analysis, we used the Mann–Whitney U test to compare rank means across demographic and clinical characteristics, with results summarized in Table 3.

The DASS-21, SF-12 and stigma questionnaire results are summarized in Table 4.

The distribution of the stigma statement responses are summarized in Table 5.

In the univariate analysis, the following associations with PSQI total score were found as significant: age (median difference 1.10; Cl 95% 0.10/2.11; *p* = 0.031); education (median difference −1.50; Cl 95% −2.50/−0.50; *p* = 0.003); employment status (median difference −1.26; Cl 95% −2.40/−0.12; *p* = 0.030); HIV-RNA < 50 copies/mL (median difference 10.82; Cl 95% 8.45/13.20; *p* = <0.001); self-report judgment on state of health (median difference −6.66; Cl 95% −7.23/−6.10; *p* = <0.001); adherence to ART (median difference −6.05; Cl 95% −6.74/−5.36; *p* = <0.001); physical activity (median difference change −6.79; Cl 95% −7.28/−6.31; *p* = <0.001); DASS-21 Depression (median difference 7.74; Cl 95% 6.69/8.79; *p* = <0.001); DASS-21 Anxiety (median difference 12.27; Cl 95% 11.23/13.31; *p* = <0.001); DASS-21 Stress (median difference 7.84; Cl 95% 6.77/8.92; *p* = <0.001); SF-12 Physical (median difference 13.16; Cl 95% 11.06/15.26; *p* = <0.001); SF-12 Mental (median difference 7.27; Cl 95% 5.93/8.61; *p* = <0.001); Stigma (median difference 6.74; Cl 95% 5.39/8.09; *p* = <0.001).

Eight multivariate analysis models were conducted for the following variables: stigma, DASS-21 Depression, DASS-21 Anxiety, DASS-21 Stress, SF-12 Physical and SF-12 Mental, Health Status, Adherence and Physical Activity. The results of the multivariate analyses are shown in Table 6 and Table 7.

Multivariate regression models estimated mean changes in PSQI total score based on predefined reference groups. For categorical predictors, the mean change is interpreted in relation to the reference category (e.g., male vs. female, low vs. high stigma). For continuous predictors, the estimate reflects the variation associated with a one-unit increase in the variable.

In the multivariate analysis models proposed, a higher mean in the PSQI total score was significantly associated with all the main variables under investigation: stigma (mean change 1.10), DASS-21 Depression (mean change 6.20), DASS-21 Anxiety (mean change 12.15), DASS-21 Stress (mean change 6.24), SF-12 Physical (mean change 7.54), SF-12 Mental (mean change 3.56), Health Status (mean change −6.04), ART Adherence (mean change −5.08) and Physical Activity (mean change −6.20) scores.

To assess the adequacy of the multivariate model, we calculated model fit indices. The R^2^ value was 1.00, indicating that the model explains all the variance in the dependent variable, though this result may be influenced by collinearity. The Akaike Information Criterion (AIC) was −259.54, and the Bayesian Information Criterion (BIC) was −261.50, suggesting a well-fitted model. However, the Adjusted R^2^ could not be computed due to collinearity issues, reinforcing the need for caution in interpreting these findings.

Multicollinearity was evaluated using the Variance Inflation Factor (VIF). The initial analysis revealed high collinearity (VIF > 10) among DASS−21 Depression, Anxiety and Stress, suggesting that these variables share substantial variance. To address this issue, we applied Principal Component Analysis (PCA) and created a single composite score, the Psychological Distress Factor, which successfully reduced collinearity. However, residual collinearity was observed among Stigma, SF-12 Physical, SF-12 Mental and Health Status. Despite these associations, we retained these variables in the model given their conceptual relevance.

## 4. Discussion

The aim of our study was to assess sleep quality, measured with the Italian version of the PSQI [29], in a sample of Italian PLWH on ART and to examine its interaction with mental status, quality of life and internalized stigma.

Good sleep quality is an indicator of well-being in PLWH [38]. In this population, poor sleep quality can be linked to difficulties in the physical and mental health spheres, poor quality of life and the experience of internalized stigma [25,39,40], and not only side effects of antiretroviral therapy or immunological dysfunction [41].

More than half of our sample reported altered habitual sleep efficiency (i.e., the difference between the number of hours spent in bed and the actual number of hours of sleep). A good percentage of patients reported high sleep latency (i.e., the time needed to fall asleep) and sleep duration, with daily dysfunction caused by altered sleep architecture. The PLWH also experienced milder difficulties with the other PSQI components (i.e., subjective sleep quality, sleep disturbance and use of sleep medication). Similarly, in Adane’s study [37] the altered PSQI components in PLWH were found to be sleep latency and habitual sleep efficacy. Chen’s study [42] reported a difference in sleep architecture between HIV+ patients and HIV-negative but sleep-disordered patients; PLWH showed a more prolonged REM phase, a lower rate of sleep-disordered breathing and a prevalence of nocturia two-fold higher compared to controls.

In addition, we found that patients who never engaged in physical activity or have done so occasionally reported poor sleep quality. Studies in the literature show how sedentary habits, such as not engaging in regular physical activity, can exacerbate the sleep disturbances of PLWH [43,44]. In Yan’s study [45], exercise was found to help reduce the sleep disturbances of HIV patients. In Vicente’s study [46], HIV patients who failed to exercise during the social distancing imposed by anti-COVID norms reported worse sleep quality.

As regards clinical and demographic characteristics, we found that middle-aged patients with a lower level of education, who were unemployed and with a viremia above 50 copies/mL reported poor sleep quality. Studies in the literature show how low education can exacerbate the sleep disturbances of PLWH [43,44]. Najafi’s study [47] reported that unemployed PLWH were twice as likely to develop sleep disorders compared to employed. Our association between a high viral load and sleep disturbances finds discordance in the literature. In some studies, sleep disturbances find association with fatigue, a very common and debilitating symptom in PLWH [48], rather than HIV-related factors such as CD4 count or viral load [49,50]. Regarding age, studies in the literature correlate sleep disorders with a younger age, precisely because younger people are much more likely to have a lower level of education because they are still in training or are unemployed [51].

Also, we observed that PLWH who have more compromised physical and mental health status and poor adherence to ART manifested poor sleep quality, in accordance with previous studies showing that insomnia can be associated with an increase in viral load through the mediation of low adherence [17,52,53]. It is likely that the action of the virus, which is not controlled by medication, reduces sleep quality, because uncontrolled HIV infection is associated with increased systemic levels of proinflammatory cytokines that lead to the development of sleep disturbances [54,55].

Our main result is the association that emerged between a high level of sleep disturbances and more symptoms of depression, anxiety, stress and internalized stigma. Furthermore, a negative correlation emerged between sleep disturbances and quality of life, i.e., as sleep disturbances increase, the score on the physical and mental SF-12 scale decreases, thus indicating worse quality of life.

Our findings confirmed previous data available in the literature. According to Adane’s study [37], PLWH with symptoms of depression and anxiety have poorer sleep quality than PLWH without these symptoms. This might be due to their reduced serotonin levels, which negatively affects sleep architecture. Other studies [53,56] have shown that patients with a more recent diagnosis of the disease are more likely to experience depressive symptoms and poor sleep quality. In Najafi’s study [47], it was found that stress negatively affects the sleep quality of HIV patients. There seems to be a strong link between stress, depression, anxiety, deficient sleep architecture and disturbances in daily life caused by poor sleep quality [17].

In particular, stress and anxiety are among the most common causes of sleep disorders, especially insomnia, in PLWH [57]. In the long term, stress and anxiety can reduce the quality of life of PLWH by reducing the activity of the immune system and accelerating disease progression [58].

As regards internalized stigma, our finding is in line with that of Bedaso et al. [17], i.e., that PLWH with poor social support are more prone to poor sleep quality. Having a safe environment where they feel protected and do not have to defend themselves against others could promote better sleep quality [59]. In fact, Hatzenbuehler [60] suggested that internalized stigma affects the sphere of health by creating interpersonal problems and increasing ruminative thought processes with negative patterns of self-perception and difficulty in approaching others. For example, patients may experience fear of revealing their HIV status to others, or fear that others will discover their HIV, fear of infecting their sexual partners and establishing new interpersonal relationships and facing changes in their own personal life [58,61].

Taken together, our data highlight the importance of the sleep disorders in evaluating psychosocial variables in addition to clinical and demographic factors.

Our study has some limitations. Firstly, it is a cross-sectional study. Therefore, we cannot establish a definite direction in associating poor sleep quality with symptoms of depression, anxiety, stress, mental and physical health and internalized stigma. A longitudinal study is needed to monitor this association over time. Second, the PSQI is a self-report questionnaire and thus can be affected by biases from some biases during compilation. Patients who are filling out the questionnaire may avoid providing some answers because of social desirability or may not realize their problems. Therefore, the study should be repeated and should include an objective method for investigating sleep quality. Third, any future study should consider the possibility of including a control group of HIV-negative patients to be able to highlight the actual differences in sleep architecture between HIV+ patients and other patients with chronic diseases. One additional limitation of our study is that we did not directly measure family and social support, which could play a crucial role in moderating the relationship between sleep disturbances, mental health, and quality of life in PLWH. Future research should consider including this variable to better control for sample variances and provide a more comprehensive understanding of psychosocial influences on sleep quality. In addition, our multicollinearity assessment using the Variance Inflation Factor (VIF) confirmed that DASS-21 Depression, Anxiety and Stress were highly intercorrelated. To mitigate this, we applied Principal Component Analysis (PCA) to create a single Psychological Distress Factor, improving the stability of the model. However, Stigma, SF-12 Physical, SF-12 Mental and Health Status still showed moderate collinearity, which should be carefully considered in future analyses. Alternative approaches, such as structural equation modeling (SEM) or hierarchical modeling, could further refine the estimation of these associations.

Finally, our patient sample primarily included virologically suppressed patients with a low percentage of comorbidities. Any future study should include patients who have a worse viro-immunological condition and other comorbidities in order to show any differences in the quality of sleep described by the patients. In any case, a well-controlled HIV-infected sample is more representative of the current Italian HIV-infected population.

## 5. Conclusions

Sleep disturbances are very common in PLWH and are associated with mental health, quality of life and internalized stigma. Possible interventions range from increasing social support to carrying out a training program to improve sleep hygiene, to work on self-perception and activities that can reduce anxiety and stress [42]. Self-acceptance interventions and the implementation of positive coping strategies are also necessary in order to reduce the negative impact of the internalized stigma on quality of life and therapeutic adherence.

## Figures and Tables

**Table 1:** Demographic and clinical characteristics of people living with HIV (n = 250).

Variables	N (%)
Sex	
Female	77 (30.8)
Male	173 (69.2)
Age (range)	
18–40	46 (18.4)
41–60	124 (49.6)
>60	80 (32.0)
Education	
Lower secondary school	53 (21.2)
Upper secondary school	128 (51.2)
Bachelor’s degree	69 (27.6)
Employment status	
Unemployed	58 (23.2)
Employed	154 (61.6)
Retired	38 (15.2)
Years from HIV diagnosis	
1–5 years	39 (15.6)
6–10 years	38 (15.2)
>10 years	173 (69.2)
Time of starting first ART regimen	
1–5 years	52 (20.8)
6–10 years	38 (15.2)
>10 years	160 (64.0)
Self-reported health status	
Poor	52 (20.8)
Good	125 (50.0)
Excellent	73 (29.2)
HIV-RNA < 50 copies/mL	
Yes	232 (92.8)
No	18 (7.2)
Self-reported adherence to ART	
Poor	31 (12.4)
Good	99 (39.6)
Excellent	120 (48.0)
Physical Activity	
Never	50 (20.0)
Occasional	115 (46.0)
Regular	85 (34.0)

Abbreviations: N—number; ART—antiretroviral therapy.

**Table 2:** The Italian version of the Pittsburgh Sleep Quality Index (PSQI) components (n = 250).

Variables	N (%) or Mean * (SD)
PSQI Component I (Subjective sleep quality)	
0 (No difficulty)	44 (17.6)
1 (Little difficulty)	142 (56.8)
2 (Moderate difficulty)	43 (17.2)
3 (Greatest difficulty)	21 (8.4)
PSQI Component II (Sleep Latency)	
0 (No difficulty)	51 (20.4)
1 (Little difficulty)	74 (29.6)
2 (Moderate difficulty)	53 (21.2)
3 (Greatest difficulty)	72 (28.8)
PSQI Component III (Sleep duration)	
0 (No difficulty)	133 (53.2)
1 (Little difficulty)	59 (23.6)
2 (Moderate difficulty)	26 (10.4)
3 (Greatest difficulty)	32 (12.8)
PSQI Component IV (Habitual sleep efficiency)	
0 (No difficulty)	54 (21.6)
1 (Little difficulty)	41 (16.4)
2 (Moderate difficulty)	24 (9.6)
3 (Greatest difficulty)	131 (52.4)
PSQI Component V (Sleep disturbances)	
0 (No difficulty)	5 (2.0)
1 (Little difficulty)	197 (78.8)
2 (Moderate difficulty)	43 (17.2)
3 (Greatest difficulty)	5 (2.0)
PSQI Component VI (Use of sleep medication)	
0 (No difficulty)	203 (81.2)
1 (Little difficulty)	6 (2.4)
2 (Moderate difficulty)	18 (7.2)
3 (Greatest difficulty)	23 (9.2)
PSQI Component VII (Daytime dysfunction)	
0 (No difficulty)	136 (54.4)
1 (Little difficulty)	76 (30.4)
2 (Moderate difficulty)	15 (6.0)
3 (Greatest difficulty)	23 (9.2)
PSQI Total *	7.84 (5.66)

Abbreviations: N—number; SD—standard deviation. For variables with an asterisk, the mean is indicated and in brackets the standard deviation. For variables without an asterisk, the frequency is indicated.

**Table 3:** Univariate analysis results.

Variable	Median Difference	95% CI	*p*-Value
Age	1.10	0.10/2.11	0.031
Sex	−0.45	−1.20/0.30	0.145
Education	−1.50	−2.50/−0.50	0.003
Employment status	−1.26	−2.40/−0.12	0.030
Years from HIV diagnosis	0.22	−0.50/0.94	0.645
Time from starting first ART regimen	0.18	−0.44/0.80	0.710
Self-reported health status	−6.66	−7.23/−6.10	<0.001
HIV-RNA < 50 copies/mL	10.82	8.45/13.20	<0.001
Self-reported adherence to ART	−6.05	−6.74/−5.36	<0.001
Physical Activity	−6.79	−7.28/−6.31	<0.001
DASS-21 Depression	7.74	6.69/8.79	<0.001
DASS-21 Anxiety	12.27	11.23/13.31	<0.001
DASS-21 Stress	7.84	6.77/8.92	<0.001
SF-12 Physical	13.16	11.06/15.26	<0.001
SF-12 Mental	7.27	5.93/8.61	<0.001
Stigma	6.74	5.39/8.09	<0.001

**Table 4:** DASS-21, SF-12 and stigma questionnaire results (n = 250).

Variables	N (%) or Mean * (SD)
DASS 21—Depression *	9.79 (9.55)
Normal (0–9)	144 (57.6)
Mild (10–12)	56 (22.4)
Moderate (13–20)	12 (4.8)
Severe (21–27)	9 (3.6)
Extremely Severe (28–42)	29 (11.6)
DASS 21—Anxiety *	4.78 (8.32)
Normal (0–6)	206 (82.4)
Mild (7–9)	3 (1.2)
Moderate (10–14)	9 (3.6)
Severe (15–19)	9 (3.6)
Extremely Severe (20–42)	23 (9.2)
DASS 21—Stress *	11.14 (10.75)
Normal (0–10)	155 (62.0)
Mild (11–18)	48 (19.2)
Moderate (19–26)	12 (4.8)
Severe (27–34)	24 (9.6)
Extremely Severe (35–42)	11 (4.4)
SF-12—physical health status *	54.07 (7.75)
Normal	231 (92.4)
Pathological	19 (7.6)
SF-12—mental health status *	45.16 (13.18)
Normal	186 (74.4)
Pathological	64 (25.6)
Stigma total score *	1.70 (2.01)

Abbreviations: N—number; SD—standard deviation. For variables with an asterisk, the mean is indicated and in brackets the standard deviation. For variables without an asterisk, the frequency is indicated.

**Table 5:** Distribution of stigma statement responses (n = 250).

Questions	Disagree N (%)	Agree N (%)	Neutral N (%)
Item 1: It is difficult to tell people about my HIV infection.	119 (47.6)	78 (31.2)	53 (21.2)
Item2: I hide my HIV status from others.	110 (44.0)	87 (34.8)	53 (21.2)
Item 3: I feel guilty that I am HIV-positive.	183 (73.2)	45 (18.0)	22 (8.8)
Item 4: I am ashamed that I am HIV-positive.	162 (64.8)	51 (20.4)	37 (14.8)
Item 5: Being HIV-positive makes me feel dirty.	201 (80.4)	42 (16.8)	7 (2.8)
Item 6: I sometimes feel worthless because I am HIV-positive.	207 (82.8)	31 (12.4)	12 (4.8)

**Table 6:** Multivariate analysis models: association between sleep quality and psychological and clinical variables.

Variable	Mean Change	95% CI	*p*-Value
Stigma (low vs. high)	1.10	0.81/1.38	<0.001
DASS-21 Depression (Normal vs. Pathological)	6.20	5.16/7.24	<0.001
DASS-21 Anxiety (Normal vs. Pathological)	12.15	10.80/13.51	<0.001
DASS-21 Stress (Normal vs. Pathological)	6.24	5.14/7.33	<0.001
SF-12 Physical (Normal vs. Pathological)	7.54	5.39/9.70	<0.001
SF-12 Mental (Normal vs. Pathological)	3.56	2.28/4.85	<0.001
Health Status	−6.04	−6.69/−5.39	<0.001
Adherence to ART	−5.08	−5.90/−4.26	<0.001
Physical Activity	−6.20	−6.75/−5.64	<0.001
Sex (Female vs. Male)	Not included in the model		
Years from HIV diagnosis	Not included in the model		
Time from starting first ART regimen	Not included in the model		
Self-reported health status	Not included in the model		

**Table 7:** Multivariate analysis models: association between sleep quality (total score at Pittsburgh Sleep Quality Index) and SF-12 Physical and Mental Health Status, Adherence to ART and Physical Activity.

	Multivariate Analysis SF-12 Physical and Mental			Multivariate Analysis Health Status			Multivariate Analysis Adherence to ART			Multivariate Analysis Physical Activity		
Variables	Mean Change	95% CI	*p*	Mean Change	95% CI	*p*	Mean Change	95% CI	*p*	Mean Change	95% CI	*p*
Sex (Female vs. Male)	-	-	-	-	-	-	-	-	-	-	-	-
Age, years	0.91	0.14/1.69	0.020 *	0.57	−0.048/1.19	0.071	0.58	−0.17/1.34	0.129	0.65	0.10/1.20	0.020 *
Education	−0.18	−0.88/0.52	0.616	0.37	−0.20–0.94	0.201	−0.09	−0.78/0.59	0.786	0.15	−0.35/0.66	0.553
Employment status	−1.30	−2.17/−0.42	0.004	−0.30	−1.03/0.41	0.401	−0.91	−1.78/−0.04	0.039	−0.45	−1.09/0.17	0.156
Years from HIV diagnosis	-	-	-	-	-	-	-	-	-	-	-	-
Time from starting first ART regimen	-	-	-	-	-	-	-	-	-	-	-	-
Self-reported health status	-	-	-	−6.04	−6.69/−5.39	<0.001 *	-	-	-	-	-	-
HIV-RNA < 50 copies/mL	6.49	4.49/8.49	<0.001	3.84	2.17/5.51	<0.001 *	4.03	1.91/6.14	<0.001 *	3.22	1.73/4.71	<0.001 *
Self-reported adherence to ART	-	-	-	-	-	-	−5.08	−5.90/−4.26	<0.001 *	-	-	-
Physical Activity	-	-	-	-	-	-	-	-	-	−6.20	−6.75/−5.64	<0.001 *
DASS-21 (Depression) Normal vs. Pathological	-	-	-	-	-	-	-	-	-	-	-	-
DASS-21 (Anxiety) Normal vs. Pathological	-	-	-	-	-	-	-	-	-	-	-	-
DASS-21 (Stress) Normal vs. Pathological	-	-	-	-	-	-	-	-	-	-	-	-
SF-12 Physical (Normal vs. Pathological)	7.54	5.39/9.70	<0.001	-	-	-	-	-	-	-	-	-
SF-12 Mental (Normal vs. Pathological)	3.56	2.28/4.85	<0.001	-	-	-	-	-	-	-	-	-
Stigma (low vs. high)	-	-	-	-	-	-	-	-	-	-	-	-

Abbreviations: ART—antiviral therapy; CI—confidence interval; *—statistically significant *p* value.

## Data Availability

The data that support the findings of this study are available from the corresponding author upon reasonable request due to restrictions for privacy.
